# High metabolic substrate load induces mitochondrial dysfunction in rat skeletal muscle microvascular endothelial cells

**DOI:** 10.14814/phy2.14855

**Published:** 2021-07-20

**Authors:** Camilla Hansen, Karina Olsen, Henriette Pilegaard, Jens Bangsbo, Lasse Gliemann, Ylva Hellsten

**Affiliations:** ^1^ Department of Nutrition, Exercise and Sports Cardiovascular Physiology Group Section of Integrative Physiology University of Copenhagen Copenhagen Denmark; ^2^ Department of Biology Section of Cell Biology and Physiology University of Copenhagen Copenhagen Denmark; ^3^ Department of Nutrition, Exercise and Sports Section of Integrative Physiology University of Copenhagen Copenhagen Denmark

**Keywords:** glucose, mitochondria, palmitic acid, reactive oxygen species, respirometry, vascular

## Abstract

The influence of glucose and palmitic acid (PA) on mitochondrial respiration and emission of hydrogen peroxide (H_2_O_2_) was determined in skeletal muscle‐derived microvascular endothelial cells. Measurements were assessed in intact and permeabilized (cells treated with 0.025% saponin) low passage endothelial cells with acute‐or prolonged (3 days) incubation with regular (1.7 mM) or elevated (2.2 mM) PA concentrations and regular (5 mM) or elevated (11 mM) glucose concentrations. In intact cells, acute incubation with 1.7 mM PA alone or with 1.7 mM PA + 5 mM glucose (*p* < .001) led to a lower mitochondrial respiration (*p* < 0.01) and markedly higher H_2_O_2_/O_2_ emission (*p* < 0.05) than with 5 mM glucose alone. Prolonged incubation of intact cells with 1.7 mM PA +5 mM glucose led to 34% (*p* < 0.05) lower respiration and 2.5‐fold higher H_2_O_2_/O_2_ emission (*p* < 0.01) than incubation with 5 mM glucose alone. Prolonged incubation of intact cells with elevated glucose led to 60% lower (*p* < 0.05) mitochondrial respiration and 4.6‐fold higher H_2_O_2_/O_2_ production than incubation with 5 mM glucose in intact cells (*p* < 0.001). All effects observed in intact cells were present also in permeabilized cells (State 2). In conclusion, our results show that acute and prolonged lipid availability, as well as prolonged hyperglycemia, induces mitochondrial dysfunction as evidenced by lower mitochondrial respiration and enhanced H_2_O_2/_O_2_ emission. Elevated plasma substrate availability may lead to microvascular dysfunction in skeletal muscle by impairing endothelial mitochondrial function.

## Graphical abstract

This study investigated and compared the impact of glucose and palmitic acid on mitochondrial respiration and emission of hydrogen peroxide measured simultaneously in low passage microvascular endothelial cells derived from rat skeletal muscle. The study shows for the first time that acute and prolonged lipid availability and prolonged hyperglycemia, as seen in lifestyle‐related diseases, lower mitochondrial respiration and enhance hydrogen peroxide emission in primary microvascular endothelial cells.

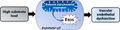

## 1 INTRODUCTION

Cardiovascular disease is one of the most common causes of death in the western world and closely associated with lifestyle (Pedersen & Saltin, 2006) and high metabolic substrate levels in plasma, i.e. hyperglycemia (Pistrosch et al., 2011) and hyperlipidemia (Nelson, 2013). At a cellular level, dysfunction of endothelial cells is central in the development of cardiovascular disease (Cai & Harrison, 2000) through effects on the regulation of vascular tone, hemostasis and inflammation (Potente & Carmeliet, 2017). Endothelial dysfunction has been proposed to be associated with defects in mitochondrial function, in part via an increased formation of reactive oxygen species (ROS) (Groschner et al., 2012; Rossman et al., 2018; Wang et al., 2012). Although endothelial cells contain only small amounts of mitochondria compared with more active cells such as skeletal muscle cells, their mitochondria are important for cellular function, both as ATP producing organelles and by contributing molecules such as calcium, which are necessary for cell function, proliferation, and survival (Caja & Enríquez, 2017; Dutta et al., 2012; Wilson et al., 2019). And despite the low content, endothelial mitochondria are thought to be a major source of ROS in the vasculature (Park et al., 2016, 2018). A particular aspect of endothelial cells is their position in the lumen of the vessels, with direct exposure to plasma levels of glucose and fatty acids. It is recognized that both hyperglycemia and hyperlipidemia, which are common in lifestyle‐related disease, (Kluge et al., 2013) are important contributors to vascular impairments in Type 2 diabetes, obesity, and metabolic syndrome, (Bülow et al., 1990; Egan et al., 1996; Hennes et al., 1996) and strict glucose control has been shown to reduce the risk of microvascular complications in Type 2 diabetes (The Diabetes Control & Complications Trial Research Group, 1993). One of the molecular mechanisms behind the detrimental effect of elevated plasma levels of glucose and fatty acids is ROS formation (Gliemann et al., 2017; Gremmels et al., 2015; Jenkins et al., 2016; Wang et al., 2017). A few previous studies have examined the influence of metabolic substrates on the formation of the reactive superoxide ($O_{2}^{\cdot ‐}$) radical and its dismutation product, hydrogen peroxide (H_2_O_2_) in endothelial cells; however, these studies have not specifically determined mitochondria as a source (Fink et al., 2012; Jenkins et al., 2016; Nishikawa et al., 2000). Moreover, there is to date no data in the literature from direct respirometry with parallel measurements of ROS in microvascular endothelial cells. In addition, the majority of the previous studies have used large vessel endothelial cells, e.g. from aorta and umbilical vein, and all have used cells at a high number of passages (Broniarek et al., 2016; Du et al., 2006; Dymkowska et al., 2017; Fink et al., 2012; Nishikawa et al., 2000), e.g. between 5 and 12. Recent studies using single‐cell transcriptomics have shown that endothelial cells exhibit similarities across tissues but less so across vessel type (Kalucka et al., 2020) and there are only few genes that are expressed in endothelial cells throughout the vascular network (Minami & Aird, 2005). Functionally, endothelial cells from different parts of the vascular tree display differences in aspects such as hemostasis, proliferative potential, and hemostasis (Aird, 2012) and evidence from the pulmonary circulation suggests that there are differences in endothelial cell metabolism in macro‐ versus microcirculation (Lee et al., 2017). In addition, although endothelial cells from different tissues may display similarities (Kalucka et al., 2020), skeletal muscle microvascular endothelial cells differ from those of most other tissues in that the microcirculation is more plastic and readily adapts both in function and growth according to the degree of muscle activity (Egginton, 2009; Hellsten & Nyberg, 2016). It is, thus, likely that the metabolic properties of skeletal muscle‐derived microvascular endothelial cells differ from that of endothelial cells from larger vessels and other organs.

The aim of the present study was to determine respiration and emission of ROS in mitochondria of primary skeletal muscle‐derived microvascular endothelial cell and, specifically, to evaluate the influence of acute and prolonged elevated metabolic substrate levels on mitochondrial function. The rationale was that thehigher substrate levels would lead to substrate overload in the mitochondriaresulting in increase in reducing pressure within the respiratory chain and increased ROS formation (Fisher‐Wellman & Neufer, 2012). It was hypothesized that prolonged exposure to elevated substrate levels would lead to a change in mitochondrial function, as indicated by reduced mitochondrial respiration and increased formation of ROS.

## 2 MATERIALS AND METHODS

### 2.1 Cell culture

**FIGURE 1:**
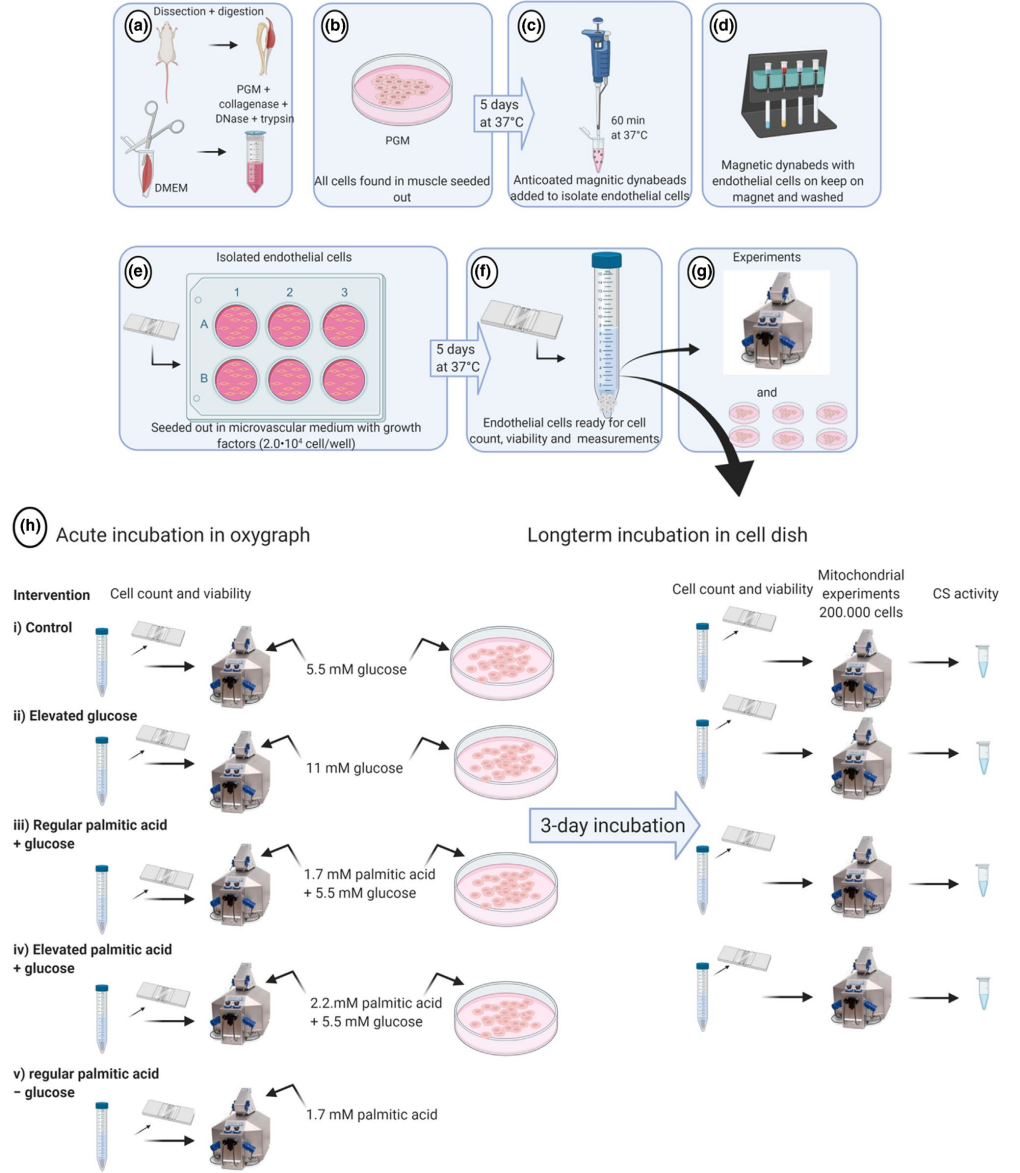
Overview of the isolation procedure and cell experiment. Upper panel show the isolation procedure (elaborated in the text) and bottom panel show the cell experiment protocol and mitochondrial measurement. (a) Depicts rat skeletal muscles from hind‐ and forelimbs were minced with scissors in Dulbecco's modified Eagle medium (DMEM) containing 1% Pen Strep and digested with 0.2% collagenase type II 0.2% collagenase, 0.01% DNase, and 0.25% trypsin. (b) Shows the single cell solution seeded out onto 110‐mm dishes for 5 days before the dynabeading procedure was proceeded. (c) Anticoated magnetic dynabeads added to the single cell solution to isolate endothelial cells. (d) A magnet was applied to collect the bound microvascular endothelial cells. (e) Cells were counted and seeded onto 35‐mm dishes. (f) Cells were confluent and ready for experiments after 4–5 days. (g) The cells were used for determination of mitochondrial respiration and H_2_O_2_ emission in acute and longterm metabolic substrate levels. (h) Overview of the diffrent acute and longterm metabolic interventions.

Skeletal muscle microvascular endothelial cell cultures were prepared from male Sprague–Dawley Rats (Taconic M&B A/S). Treatment of animals was approved by Danish National Animal Experiments Inspectorate. Animals had ad libitum food and water available until termination. Ten 100 g rats were used in total. After cervical dislocation, all muscles from hind‐ and forelimbs were removed and placed on ice in Dulbecco's phosphate buffered saline (DPBS) (Life Technologies) with 1% glucose +1% pencillin‐streptomycin (Pen Strep) (Life Technologies). The muscle tissue was minced with scissors and digested with 0.2% collagenase type II (Worthington Biochemicals) in Dulbecco's modified Eagle medium (DMEM) (Life Technologies) containing 1% Pen Strep for 1.5 h at 37°C under rotation (Figure 1a). Afterwards, the suspension was centrifuged, the pellet was incubated under rotation in a solution of 0.2% collagenase, 0.01% DNase (Sigma‐Aldrich), and 0.25% trypsin (Life Technologies) in DMEM containing 1% Pen Strep for 30 min at 37°C.

To stop trypsinization reaction, ice‐cold primary growth medium (PGM) [DMEM supplemented with 1% Pen Strep, horse serum (HS, 10%), and fetal bovine serum (FBS, 10%) (Life Technologies) were added to the suspension and centrifuged at 350*g* for 10 min. The supernatant was removed; the pellet was passed through a 70 µm filter and washed with PGM. The suspended cells were counted and seeded out onto 110‐mm dishes (0.9 × 10^6^ cells/dish) coated with attachment factor (s‐006‐100, Gibco, ThermoFisher Scientific) (Figure 1b). PGM was used for the initial seeding of all cell types, but for the below isolation and further culture of endothelial cells a low serum growth factor medium (111–500, Cell Applications Inc.) was used.

After 5 days in culture, the microvascular endothelial cells from skeletal muscle were harvested by using trypsin/EDTA solution (Sigma‐Aldrich). The pellet was resuspended in media containing dynabeads (Invitrogen) coated with Griffonia Simplifolica lectin (B‐1105, Vector laboratories) and an antibody to FLK‐1 (VEGF receptor 2, Flk‐1 (C‐1158, Santa Cruz biotechnology), respectively (Figure 1c). The cell suspension was incubated for 60 min at 37°C. A magnet was applied to collect the bound microvascular endothelial cells (Figure 1d). Cells were washed, resuspended, and cultured in microvascular medium (111–500, Cell Applications Inc.) with microvascular growth supplement (111‐GS, Cell Applications Inc.) containing 5% FBS serum, 5 mM glucose, and range of amino acids including l‐arginine and l‐glutamine. Cells were counted and seeded onto 35‐mm dishes (2 × 10^4^ cells/dish) coated with attachment factor protein (Figure 1e). Cells were confluent and ready for experiments after 4–5 days (Figure 1f). As the endothelial cells were isolated from several muscle groups with different fiber type compositions, the cell origin was from a mixture of muscle fiber types.

### 2.2 Cell experiment

On the experimental day, the cells were starved with 0.1% BSA in medium 1 h before harvest (Potente & Carmeliet, 2017; Zecchin et al., 2017). The microvascular endothelial cells were harvested with trypsin/EDTA after first or second passage. The cells were used for determination of mitochondrial respiration and H_2_O_2_ emission in the presence of different metabolic substrate levels where the concentrations of substrates used were physiologically relevant, i.e. resembling concentrations seen in plasma in conditions of healthy versus lifestyle‐related disease (Abdelmagid et al., 2015; Bonora et al., 2001; Feng et al., 2017). The cells were suspended in z‐buffer and mitochondrial respiration, and H_2_O_2_ emission was determined in the presence of (i) regular glucose levels (5 mM) Control; (ii) elevated glucose levels (11 mM) (Bonora et al., 2001); (iii) regular palmitic acid concentration (1.7 mM) (Abdelmagid et al., 2015) combined with regular glucose levels or; (iv) elevated palmitic acid concentration (2.2 mM) (Feng et al., 2017) combined with regular glucose levels (Figure 1g,h). In a second set of experiments, the cells were seeded out and cultured to 70% confluence whereafter they were incubated by adding the same substrate to the media as described in the acute trial (i–iv) but for 3 days. After the 3 days of incubation, the cells were confluent and rinsed with DMEM and incubated with 37°C microvascular medium with growth factor for 3 h prior to measurements. Measurements after the 3‐day incubations were conducted in z‐buffer under control conditions (5 mM glucose). Palmitic acid was diluted in 100% DMSO, and final DMSO concentration in the cell medium (containing albumin to get albumin‐conjugated palmitate) was <1%. DMSO was also used in the control cells. In addition, the effect of DMSO on cell viability, mitochondrial respiration, and ROS formation were evaluated in a pilot trial and found not to influence the outcome (data not shown).

### 2.3 Cell viability

To asses cell viability, equal amount cell solution with PBS as media and trypan blue 0.4% (15250061; Gibco, Thermo Fischer Scientific) were mixed, and measurements were made in a hemocytometer. Viability is expressed as percentage of viable cells relative to total number of cells (Strober, 2015). No difference in cell viability was found between the four interventions.

### 2.4 Measurement of mitochondrial respiration and H_2_O_2_ emission

Mitochondrial respiration and H_2_O_2_ emission were measured simultaneously in ~200,000 intact and permeabilized rat microvascular endothelial cells in a respirometer (Oxygraph‐2k; Oroboros). All measurements were achieved at atmospheric oxygen levels (approximately between 200 and 100 nmol O_2_ in the chamber) at 37°C. The respiration was measured in buffer Z containing EGTA (1 mM); MgCl_2_ (5 mM); K^+^‐MES (105 mM); KCl (30 mM); KH_2_PO_4_ (10 mM); and albumin (5 mg/ml) at pH 7.1 (Perry et al., 2011). The protocol used was a *Substrate*‐*Uncoupler*‐*Inhibitor Titration* *(SUIT)* protocol (Gnaiger, 2014) (Figure S1) and consisted of (all substrate concentrations are final concentrations) exogenous superoxide dismutase (SOD; 5 U/ml) (Sigma‐Aldrich, MERCK) + Amplex Ultra Red (AmR; (10 µM) (Thermofischer), and Horse Radish Peroxidase (HRP) (Sigma‐Aldrich, Merck) (1 U/ml). Measurements were made in both intact and permeabilized cells; in intact cells to provide physiologically relevant measures and, in permeabilized cells, to evaluate mitochondrial respiration and H_2_O_2_ emission at the different complexes. Glucose and/or palmitic acid were added as described above to investigate the influence of metabolic substrates in intact cells. In prolonged experiments, respiration and ROS emission were measured in the presence of 5 mM glucose for all conditions in order to separate out the effect of long‐term versus acute substrate availability. To achieve permeabilization, saponin (0.025%) was added to in the respirometer to permeabilize cells for 10 min before other substrates were added to the chamber. Then glutamate (10 mM) + malate (2 mM) and succinate (1 and 10 mM) (state 2) were titrated in two steps, followed by saturating ADP (5 mM) + magnesium (Mg; 5 mM) (state 3). Thereafter, oligomycin (2.5 µM) was added to inhibit the ATP synthase (state 4o), FCCP (0.5 µM) titrated stepwise to obtain maximum mitochondrial respiration in the uncoupled state (Pesta & Gnaiger, 2012) (State 3u). Rotenone (0.5 µM) was added to inhibit Complex I and measure complex II‐linked respiration. Antimycin A (2.5 µM) was then added to inhibit complex III and infer non‐mitochondrial respiration. Mitochondrial membrane integrity was assessed with cytochrome C in separate experiments (*n* = 3). The results showed less than 1% change in respiration, indicating that cryopreservation, sample preparation, and the saponin treatment did not affect the integrity of the outer mitochondrial membrane.

### 2.5 H_2_O_2_ determination

Every step was separated by a H_2_O_2_ (0.1 µM) calibration to take into account that the sensitivity of fluorometric sensor changes with time. The determination of H_2_O_2_ is based on oxidation of H_2_O_2_ with AmR to a red fluorescent compound, resurofin, catalyzed by the enzyme HRP (Krumschnabel et al., 2015; Mohanty et al., 1997). The H_2_O_2_ emission was determined fluorometrically at the excitation wavelength (560 nm) and emission wavelength (590 nm). Sensitivity decline of the resurofin reaction was calculated in every experiment as the delta change in emission intensity of the autoxidation in buffer Z compared with the delta change in emission intensity in presence of all substrates titrated and cells. The H_2_O_2_ emission was calculated from the slope (pmol/(s·ml)) relative to the sensitivity decline and expressed relative to amount of viable endothelial cells (described in section cell viability) in the chamber.

### 2.6 Flux control ratio (FCR) and leak‐control ratio

The FCR is a ratio of oxygen flux in different respiratory control states normalized to maximum flux in the SUIT protocol as reference state to obtain a theoretical lower an upper limit of 0.0 to 1.0 (0% and 100%) (Pesta & Gnaiger, 2012). FCR provides an indication of coupling control and mitochondrial efficiency (Doerrier et al., 2018). The leak‐control ratio is the flux ratio of the leak respiration normalized to maximal phosphorylation capacity measured and is an indication of uncoupling of the mitochondria at constant OXPHOS capacity (Pesta & Gnaiger, 2012).

### 2.7 Immunocytochemistry

To assess purity of the cell cultures, the isolated microvascular endothelial cells were seeded out onto glass slides, fixed with 2% formaldehyde for 10 min (Sigma Aldrich, Merck) and stained for DNA with DAPI (Vector laboratories) and with the endothelial specific lectin Griffonia Simplifolica lectin (B‐1105, Vector laboratories).

### 2.8 CS activity

CS activity was used as an indication of mitochondrial content. CS has been validated as strong predictor of mitochondrial content in skeletal muscle(Larsen et al., 2012) and has also been used in endothelial cell cultures and arteries (Broniarek et al., 2016; Park et al., 2018). Cells were lysed in a 0.3 mol/L phosphate BSA buffer adjusted to pH 7.7 and analyzed for the maximal enzyme activity of citrate synthase (CS) using a fluorometric method (Fluoroscan Ascent, Thermo Scientific), as previously described (Lowry, 1972).

### 2.9 Statistical analyses

Statistical analyses were performed with R (version 3.4.1; R Foundation for Statistical Computing) using the interface RStudio (version 1.1.463; RStudio Team). One‐way ANOVA and Tukey multiple pairwise comparison were used to compare the differences in the mitochondrial states of mitochondrial respiration and ROS emission in presence of regular glucose for endothelial cells with mitochondrial blockers and control media. For the rest of the analysis, a linear mixed model was performed to investigate differences between interventions and within intervention (different mitochondrial steps). Fixed factors were “intervention” (substrate incubation [glucose vs. PA and glucose] and “group” protocol [intact cells, glucose 5 mM, LeakCI + CII (10 mM]). Differences between rats were modeled as random effects. Comparisons were made between glucose and PA and glucose interventions. The homogeneity of variance and normal distribution was confirmed through residual and Q–Q plots. A Tukey post hoc procedure was used to detect pairwise differences, performed with multi comparison and non‐adjusted p‐values are reported. Graphs were created with GraphPad Prism 6.01 (1992–2012 GraphPad Software, Inc.). The level of significance was set at *p* < 0.05 at a power level of 0.8. Tendencies are reported when 0.05 ≤ *p* < 0.1. All data are presented as mean ± SEM.

## 3 RESULTS

### 3.1 Cell purity and viability

**FIGURE 2:**
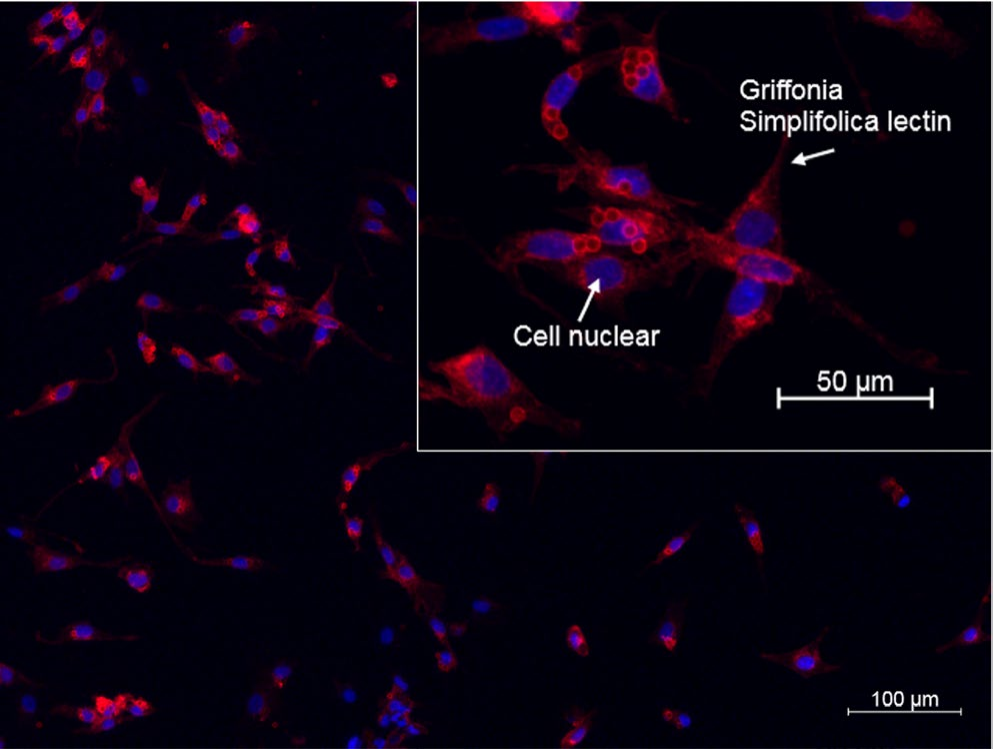
Representative micrograph showing immunohistochemical identification of microvascular endothelial cell suspension used for respirometry measurements. Arrows shows Griffonia Simplifolica lectin (red) staining for identification of endothelial cells and DAPI (blue) staining for cell nuclei. Remaining antibody‐coated magnetic dynabeads appear as circle shapes. Overall, staining procedures identified that approximately 95% of the cells were positive for the lectin

Immunocytochemical assessments of the cell batches indicated a purity of the microvascular endothelial cell cultures of over 95% (see Figure 2).

Viability of the cells was determined prior to mitochondrial measurements in the respirometer and was found to be on average 92 ± 0.5%. For the separate conditions, viability was as follows: for acute incubated cells 98 ± 0.1%; for prolonged incubated cells with regular and high glucose 93 ± 0.8 and 94 ± 1.0%, respectively; and for prolonged incubated cells with regular and high palmitic acid 90 ± 1.8%, and 89 ± 1.6%, respectively.

### 3.2 Mitochondrial respiration and ROS production in intact and permeabilized microvascular endothelial cells

**FIGURE 3:**
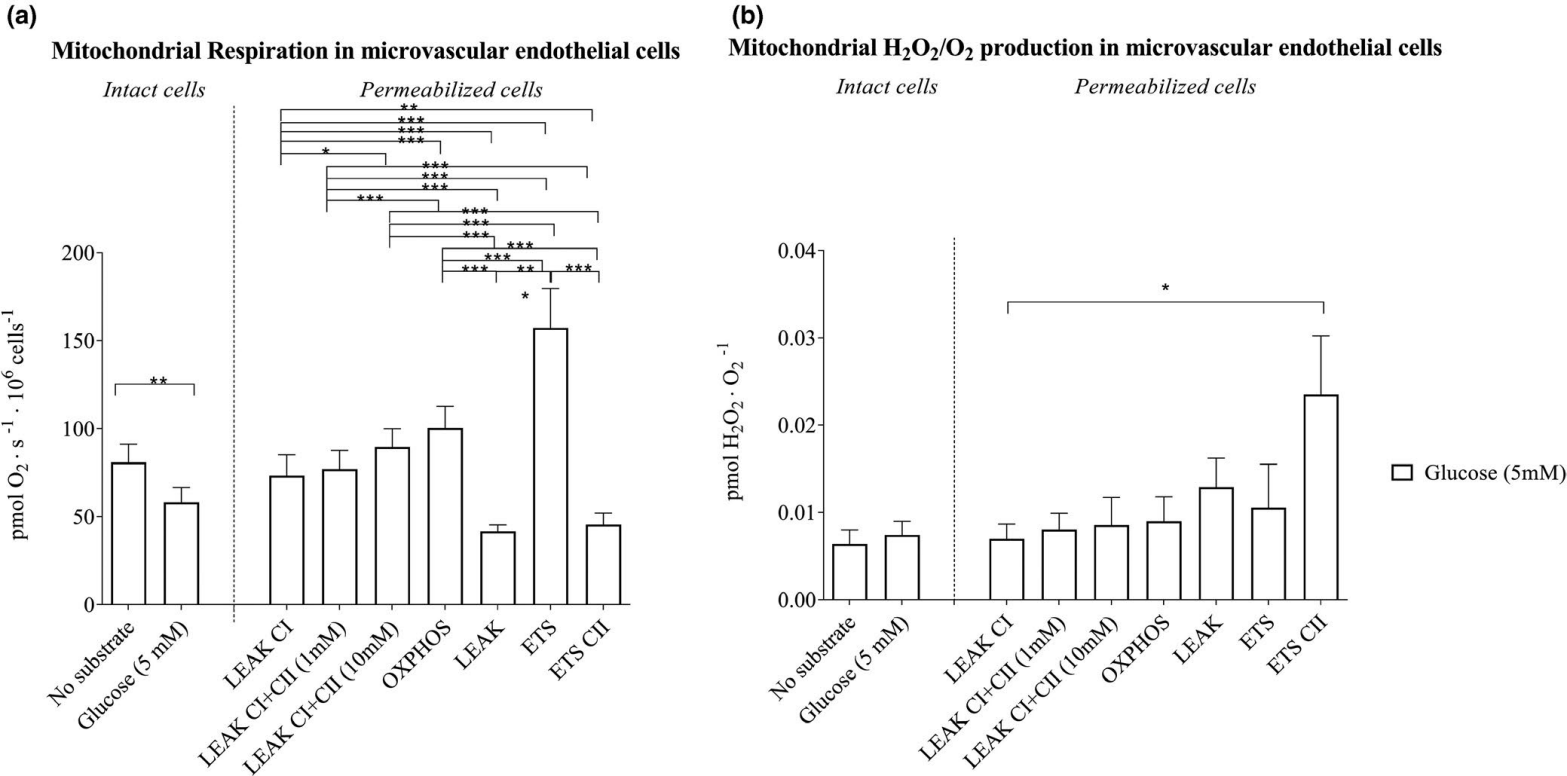
SUIT protocol of mitochondrial respiration and H_2_O_2_/O_2_ emission in microvascular endothelial cells in the presence of 5 mM glucose. (a) Mitochondrial respiration and (b) mitochondrial H_2_O_2_/O_2_ emission in intact and permeabilized cell (*n* = 10 in each state). Data was analyzed by one‐way ANOVA and Tukey multiple pairwise‐comparison. *Significant difference between states in the SUIT protocol in presence of glucose (5 mM). ^**^Denotes significant difference *p* < 0.01. ^***^Denotes significant difference *p* < 0.001. All data are presented as mean ± SEM. LEAK CI; Leak state with substrates feeding complex I (glutamate and malate), LEAK CI + II; Leak state with complex I and complex II substrates (2 mM succinate), LEAK CI + CII (10 mM); Leak state using 10 mM succinate complex I and complex II leak respiration, OXPHOS; Maximal respiration with 5 mM ADP (state 3), LEAK; Leak state with oligomycin (state 4o), ETS; Electron transfer system by uncoupling oxidative phosphorylation with FCCP, ETS CII; Complex II flux with rotenone (shutdown of complex I)

In intact cells, the mitochondrial respiration was 28% lower (*p* < 0.01) in the presence of glucose than with no substrate, while H_2_O_2_ emission was similar with and without glucose present (Figure 3). There was no influence of addition of insulin (30 pmol/L (Larsen et al., 2009)) (data not shown).

In permeabilized cells, significant differences in mitochondrial respiration were observed with every subsequent step in the SUIT protocol (*p* < 0.001; Figure 3a). There was no difference in H_2_O_2_ emission between the states (Figure 3b). A 22% decrease (*p* < 0.001) in mitochondrial respiration was observed in leak state (state4O). Moreover respiration in ETS with fully uncoupled mitochondria was higher than all other steps in the SUIT protocol than in intact cells (*p* < 0.001; Figure 3a). H_2_O_2_ emission was 2.1‐fold higher in ETS CII than in complex I supported mitochondrial respiration (*p* < 0.05; Figure 3b).

### 3.3 Mitochondrial respiration and H_2_O_2_ emission after acute incubation with glucose and palmitic acid

**FIGURE 4:**
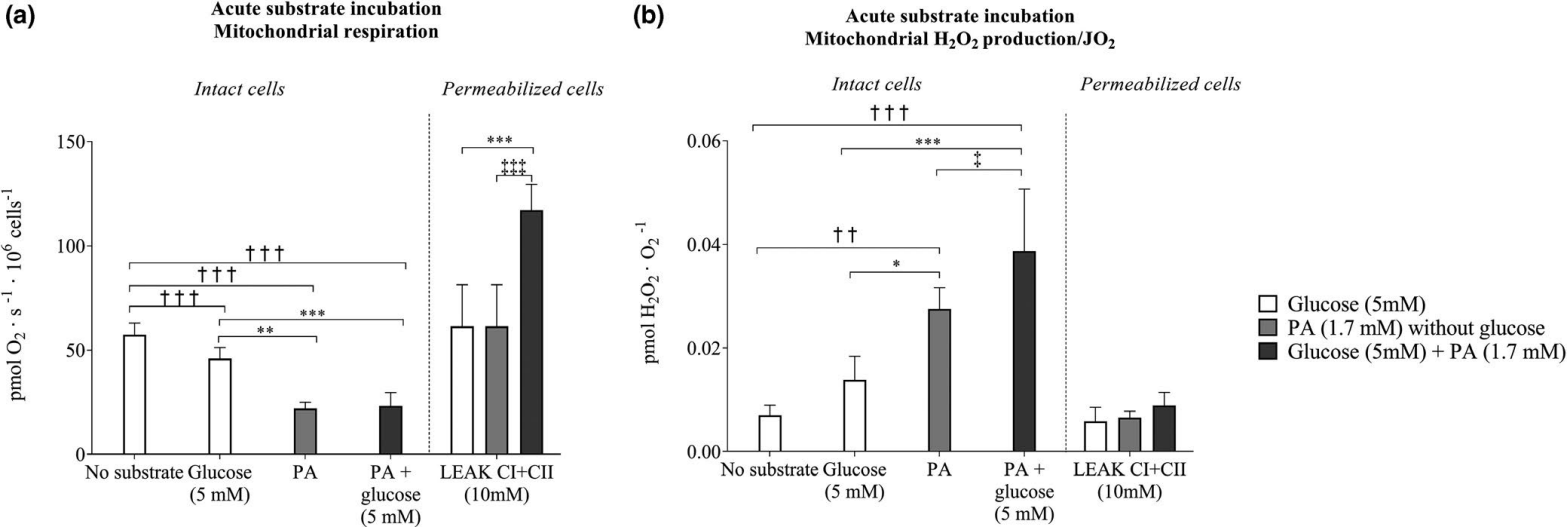
Mitochondrial respiration and hydrogen peroxide (H_2_O_2_/O_2_) emission in in intact and permeabilized microvascular endothelial cells in the presence of acute substrate. (a) Depicts mitochondrial respiration in presence of 1.7 mM palmitic acid (PA) with or without 5 mM glucose, and (b) mitochondrial H_2_O_2_/O_2_ emission in presence of 1.7 mM palmitic acid (PA) with or without 5 mM glucose (*n* = 10 in glucose intervention, *n* = 8 in glucose + PA intervention, and *n* = 5 in PA intervention). Data were analyzed with linear mixed model. *Significantly different from glucose (5 mM), *p* < 0.05. ^**^Denotes significantly different (*p* < 0.01) from glucose. ^***^Denotes significantly different (*p* < 0.001) from glucose. ^††^Denotes significantly different (*p* < 0.01) from no added substrate. ^†††^Denotes significantly different (*p* < 0.001) from no added substrate. ^‡^Significantly different from PA (1.7 mM). ^‡‡‡^Denotes significantly different (*p* < 0.001) from PA. LEAKCI + CII (10 mM); Leak state with complex I and complex II substrates (10 mM succinate) the maximal complex I and complex II leak respiration. All data are presented as mean ± SEM

In intact cells, there was 26% lower mitochondrial respiration in the presence of regular (5 mM) glucose than with no added substrate (*p* < 0.001; Figure 4a). In the presence of regular (1.7 mM) palmitic acid, mitochondrial respiration was 28% lower (*p* < 0.01; Figure 4a) than with 5 mM glucose. The H_2_O_2_/O_2_ emission was 2.1‐fold higher in the presence of palmitic acid than glucose (*p* < 0.05; Figure 4b). In the presence of 1.7 mM palmitic acid and 5 mM glucose combined, mitochondrial respiration was 36% lower than with 5 mM glucose alone (*p* < 0.001; Figure 4a). The H_2_O_2_/O_2_ emission in the presence of 1.7 mM palmitic acid 5 mM glucose combined was 45% higher (*p* < 0.05; Figure 4b) than with 1.7 mM palmitic acid alone and 3.9‐fold higher (*p* < 0.001) than with 5 mM glucose alone. All of the effects of the acute substrate incubations observed in the intact cells were present also in State II mitochondrial respiration (Figure 4).

### 3.4 Mitochondrial respiration and H_2_O_2_ emission with acute regular and elevated substrate levels

**FIGURE 5:**
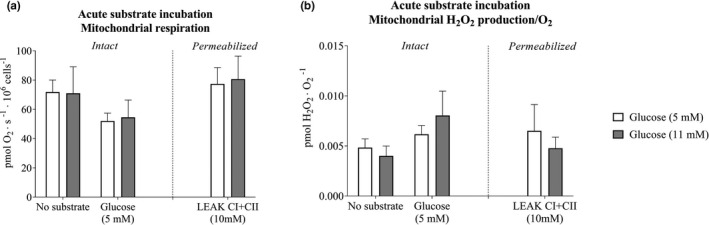
Mitochondrial respiration and hydrogen peroxide (H_2_O_2_/O_2_) emission in intact and permeabilized microvascular endothelial cells in the presence of acute glucose intervention. (a) Mitochondrial respiration in presence of regular (5 mM) (*n* = 6) or elevated (11 mM) glucose levels (*n* = 7), and (b) Mitochondrial H_2_O_2_/O_2_ emission in intact and permeabilized cells (LEAK CI + CII) in the presence of regular (5 mM) (*n* = 6) or elevated (11 mM) glucose levels (*n* = 7). Data were analyzed with linear mixed model. LEAKCI + CII (10 mM); Leak state with complex I and complex II substrates (10 mM succinate) the maximal complex I and complex II leak respiration. All data are presented as mean ± SEM

There was no difference in mitochondrial respiration or H_2_O_2_/O_2_ emission between the conditions of regular (5 mM) or elevated (11 mM) glucose in either intact or permeabilized cells (Figure 5).

**FIGURE 6:**
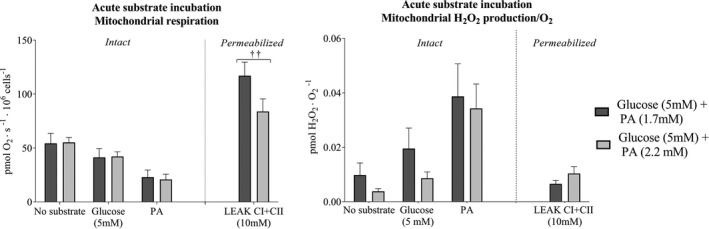
Mitochondrial respiration and hydrogen peroxide (H_2_O_2_/O_2_) emission in intact and permeabilized microvascular endothelial cells in the presence of acute palmitic acid intervention. (a) Mitochondrial respiration in the presence of regular (1.7 mM) or elevated (2.2 mM) PA concentrations and regular (5 mM) glucose, and (b) mitochondrial H_2_O_2_/O_2_ emission in intact and permeabilized cells (LEAK CI + CII) (*n* = 8 in regular PA and glucose, and *n* = 9 in elevated PA and glucose). Data were analyzed with linear mixed model. ^††^Denotes significantly different from regular PA *p* < 0.01. Leak state with complex I and complex II substrates (10 mM succinate) the maximal complex I and complex II leak respiration. All data are presented as mean ± SEM

There was no difference in mitochondrial respiration in intact cells between the presence of regular (1.7 mM) and elevated (2.2 mM) palmitic acid. In permeabilized cells, complex I+II‐supported mitochondrial respiration was 28% lower (*p* < 0.01; Figure 6a) in the presence of 2.2 mM palmitic acid than in the presence of 1.7 mM palmitic acid, with no difference in H_2_O_2_/O_2_ emission.

### 3.5 Mitochondrial respiration and H_2_O_2_ emission after 3 days of incubation with regular palmitic acid and glucose levels

**FIGURE 7:**
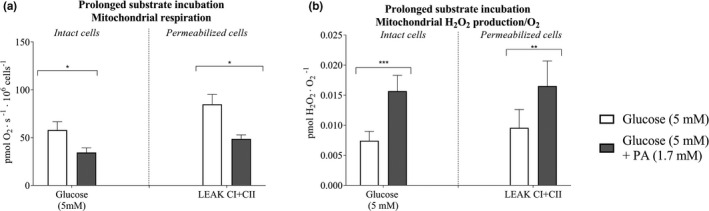
Mitochondrial respiration and hydrogen peroxide (H_2_O_2_/O_2_) emission after 3‐days prolonged glucose and palmitic acid incubation of microvascular endothelial cells. (a) Mitochondrial respiration in the presence of regular (5 mM) glucose or regular glucose and regular (1.7 mM) palmitic acid (PA), and (b) mitochondrial H_2_O_2_/O_2_ emission in intact with 5 mM glucose concentration and permeabilized cells (LEAK CI + CII) with regular (5 mM) glucose or regular glucose and regular (1.7 mM) PA incubations (*n* = 10 in glucose intervention and *n* = 6 in PA intervention). Data were analyzed with linear mixed model. *Significantly different from regular glucose (5 mM) *p* < 0.05. ** shows *p* < 0.01 and *** shows *p* < 0.001. All data are presented as mean ± SEM. LEAKCI + CII; Leak state with complex I and complex II substrates with 10 mM succinate

In intact cells, a 3‐day incubation of cells with regular (1.7 mM) palmitic acid resulted in 34% lower (*p* < 0.05; Figure 7a) mitochondrial respiration and a 2.5‐fold higher (*p* < 0.001; Figure 7b) H_2_O_2_/O_2_ emission than in cells incubated for 3 days with regular glucose.

In permeabilized cells, State II mitochondrial respiration was 35% lower (*p* < 0.05; Figure 7a) and H_2_O_2_/O_2_ was 2.0‐fold higher (*p* < 0.01; Figure 7b) with 1,7 mM palmitic acid than with 5 mM glucose incubation.

The CS activity was not different between cells incubated for 3 days with either 1.7 mM palmitic acid and 5 mM glucose combined, or with 5 mM glucose alone (Figure 10), indicating no difference in mitochondrial volume between interventions. Intrinsic mitochondrial respiration (respiration related to CS activity) in intact and permeabilized cells was 62% and 38% lower (*p* < 0.01 and *p* < 0.001), respectively, when incubated for 3 days with regular palmitic acid and glucose combined than when incubated with regular glucose concentration alone (Figure 11a).

### 3.6 Mitochondrial respiration and H_2_O_2_ emission after 3 days incubation with regular and elevated glucose concentrations

The microvascular endothelial cells were incubated in either regular (5 mM) or elevated (11 mM) glucose for 3 days, and mitochondrial measurements were conducted in the presence of regular glucose.

**FIGURE 8:**
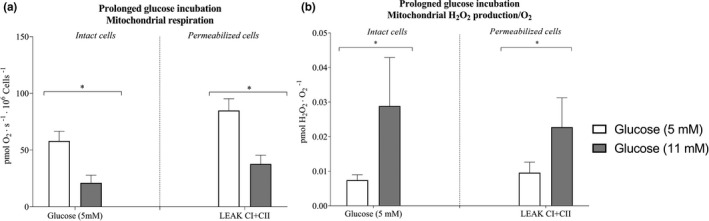
Mitochondrial respiration and hydrogen peroxide (H_2_O_2_/O_2_) emission after 3‐days prolonged glucose incubation of microvascular endothelial cells. (a) Mitochondrial respiration after 3‐days of incubation of microvascular endothelial cells with regular (5 mM) or elevated (11 mM) glucose concentrations, and (b) Mitochondrial H_2_O_2_/O_2_ emission in intact with 5 mM glucose concentration and permeabilized cells in regular and elevated glucose (LEAK CI + CII) (*n* = 10 regular‐ and *n* = 8 in elevated glucose concentration). Data were analyzed with linear mixed model. ^*^Significantly different from regular glucose (5 mM) *p* < 0.05. All data are presented as mean ± SEM. LEAKCI + CII; Leak state with complex I and complex II substrates with 10 mM succinate

Intact cells that had been incubated for 3 days with 11 mM glucose had a 60% lower respiration (*p* < 0.05; Figure 8a) and 4.6‐fold higher H_2_O_2_/O_2_ emission (*p* < 0.05; Figure 8b) than the cells incubated with 5 mM glucose concentration. In permeabilized cells, mitochondrial respiration in State II was 53% lower (*p* < 0.05; Figure 8a) and H_2_O_2_/O_2_ was 2.8‐fold higher (*p* < 0.05; Figure 8b) in cells incubated with 11 mM than with 5 mM glucose concentration. The lactate concentrations in media after 3 days were not different between the four substrate conditions.

The CS activity was not different between cells incubated for 3 days with either 11 mM glucose compared with regular glucose (Figure 10). Mitochondrial respiration normalized to CS activity was 55% lower (*p* < 0.01; Figure 11b) in intact cells and 43% lower (*p* < 0.001; Figure 11b) in permeabilized cells incubated with 11 mM glucose than when incubated with regular glucose.

### 3.7 Mitochondrial respiration and H_2_O_2_ emission after 3 days incubation with regular or elevated palmitic acid level

The endothelial cells were incubated for 3 days in either regular (1.7 mM) or elevated (2.2. mM) palmitic acid and 5 mM glucose for 3 days, and mitochondrial measurements were conducted in the presence of regular glucose concentrations.

**FIGURE 9:**
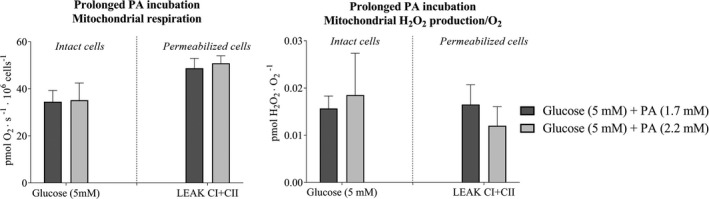
Mitochondrial respiration and hydrogen peroxide (H_2_O_2_/O_2_) emission after 3‐days prolonged palmitic acids incubation of microvascular endothelial cells. (a) Mitochondrial respiration after 3‐days incubation of microvascular endothelial cells with regular (1.7 mM) or elevated (2.2 mM) PA concentrations and regular glucose (5 mM) (*n* = 6 in each group), and (b) Mitochondrial H_2_O_2_/O_2_ emission in intact with 5 mM glucose concentration and permeabilized cells (LEAK CI + CII) in regular and elevated PA concentrations (*n* = 6 in each group). Data were analyzed with linear mixed model. All data are presented as mean ± SEM. LEAKCI + CII; Leak state with complex I and complex II substrates with 10 mM succinate

There was no difference in mitochondrial respiration or H_2_O_2_/O_2_ emission between cells incubated with regular or elevated palmitic acid concentration for 3 days, in either intact or permeabilized cells (Figure 9).

**FIGURE 10:**
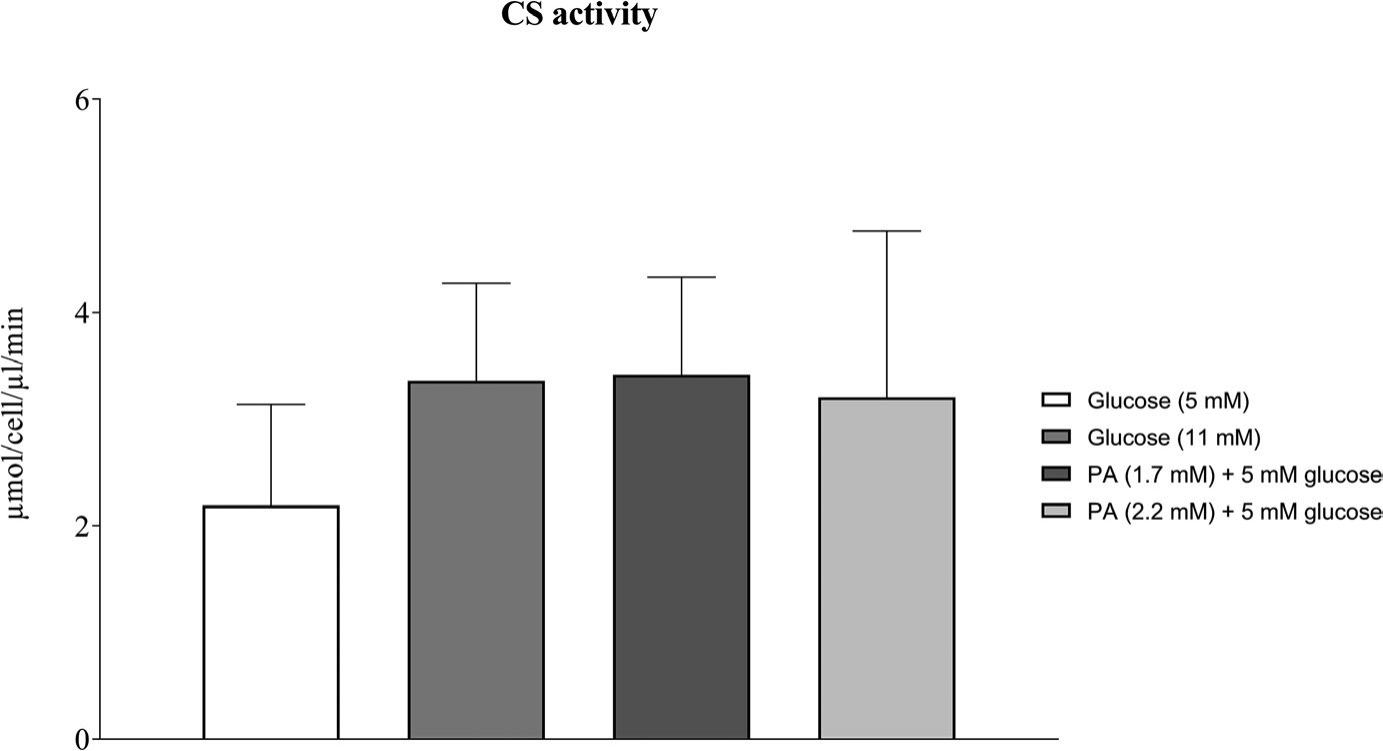
CS activity in microvascular endothelial cells after 3‐day incubation with regular and elevated glucose or regular and elevated palmitic acid (PA) with 5 mM glucose concentrations (*n* = 6 in each group). Data were analyzed with linear mixed model. All data are presented as mean ± SEM. PA; palmitic acid, Maximal oxidation is achieved using FCCP, which means maximal uncoupled conditions

**FIGURE 11:**
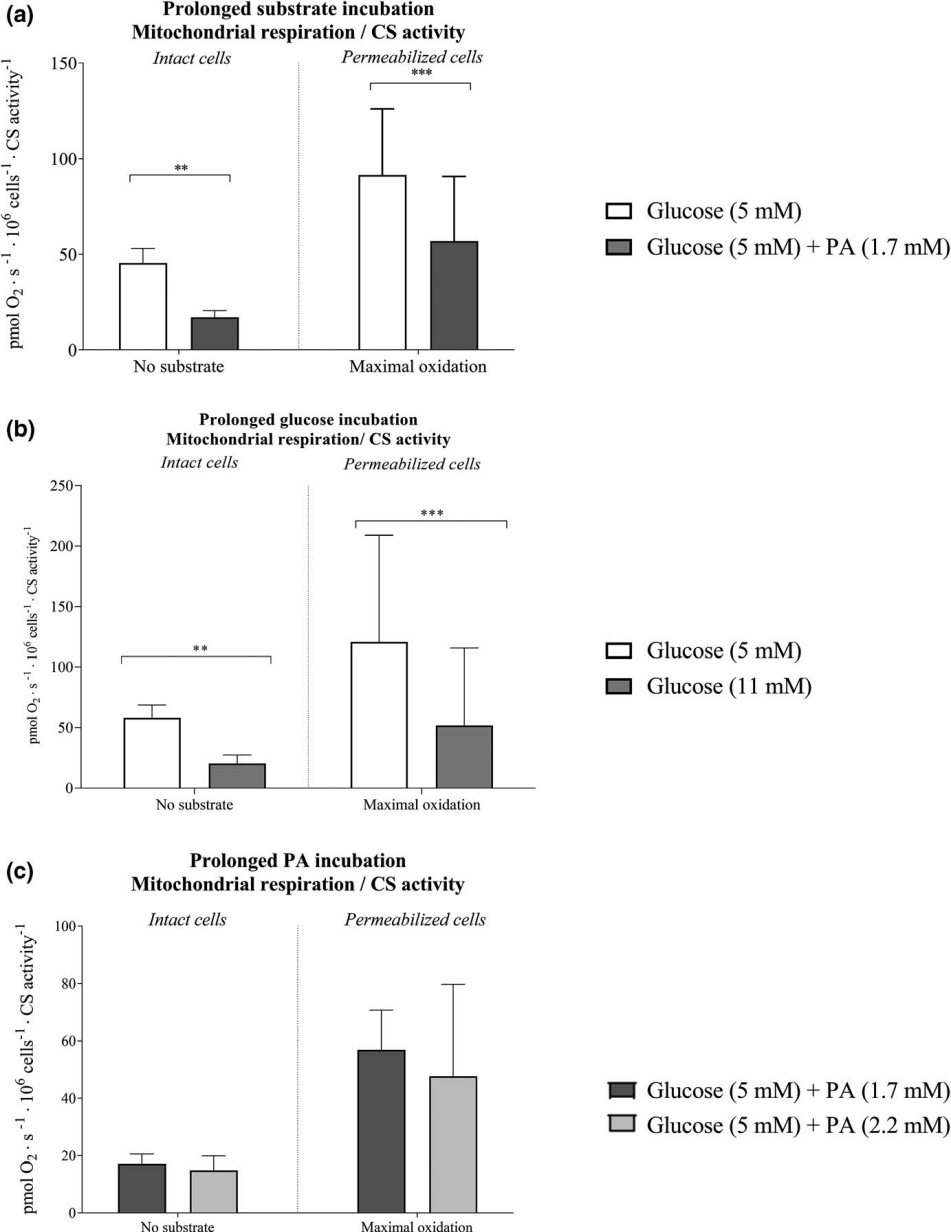
Intrinsic mitochondrial respiration in microvascular endothelial cells after 3‐day incubation with regular and elevated glucose or regular and elevated palmitic acid (PA) with 5 mM glucose concentrations. (a) Mitochondrial respiration divided by CS activity in microvascular endothelial cells after 3 days of incubation with regular glucose or regular palmitic acid levels (*n* = 6 in each group), (b) Intrinsic mitochondrial respiration after 3 days incubation with regular or elevated glucose levels (*n* = 6 in each group), and (c) Intrinsic mitochondrial respiration after 3 days incubation with regular or elevated PA levels with 5 mM glucose (*n* = 6 in each group). Data were analyzed with linear mixed model. ^**^Denotes a significantly different from regular (5 mM) glucose *p* < 0.01. ^***^Denotes significantly different from regular (5 mM) glucose *p* < 0.001. All data are presented as mean ± SEM. PA, palmitic acid; Maximal oxidation is achieved using FCCP, representing maximal uncoupled conditions

CS activity was not different between cells incubated for 3 days with either 1.7 mM or 2.2 mM palmitic acid concentration levels (Figure 10) and thereby no differnce in the intrinsic mitochondrial respiration (Figure 11c).

### 3.8 Flux control ratios

In the presence of acute incubation of regular PA without glucose, coupling efficiency was 4.2‐fold lower (*p* < 0.001) than in the presence of glucose alone. The presence of either acute regular or elevated PA combined with regular glucose coupling efficiency was 4.6‐ and 5.6‐fold lower (*p* < 0.001), respectively than with regular glucose.

When cells had been incubated for 3‐days with elevated glucose, the leak‐control ratio of the mitochondria was 3.1‐fold (*p* < 0.001) higher than control. In cells incubated for 3 days with elevated PA, leak‐control ratio was 1.5‐fold higher (*p* < 0.05) than cells incubated with regular PA concentration (Table 1).

**TABLE 1:** Flux control ratios

	Acute incubation with glucose		Acute incubation with palmitic acid (with 5 mM glucose)		Acute palmitic acid (without glucose)
	5 mM	11 mM	1.7 mM	2.2 mM	1.7 mM
LEAK/ETS	0.30 ± 0.01	0.21 ± 0.03	0.95 ± 0.22^***^	1.14 ± 0.13^***^	0.87 ± 0.20^**^
CI/CI + II	0.48 ± 0.04	0.69 ± 0.04 (*)*p* = 0.061	0.36 ± 0.07^***^	0.34 ± 0.05^***^	0.65 ± 0.17 ^††, ‡‡‡^
CI + II/OXPHOS	0.83 ± 0.03	0.68 ± 0.10	1.01 ± 0.05^***^	1.00 ± 0.03^***^	0.93 ± 0.05^*^

	Prolonged incubation with glucose		Prolonged incubation with palmitic acid (with 5 mM glucose)		
	5 mM	11 mM		1.7 mM	2.2 mM
LEAK/ETS	0.18 ± 0.03	0.56 ± 0.13^***^		0.29 ± 0.03^‡^	0.19 ± 0.03
CI/CI + II	0.81 ± 0.05	1.08 ± 0.08 kn^**^		0.66 ± 0.08	0.64 ± 0.03
CI + II/OXPHOS	0.77 ± 0.07	0.96 ± 0.07		0.61 ± 0.08^***^	0.58 ± 0.07^***^

The top table shows flux control ratios (FCR) for acute interventions. The bottom table shows FCR for prolonged interventions. Acute glucose (*n* = 10), acute PA with 5 mM glucose (*n* = 8), acute palmitic acid without glucose intervention (*n* = 5), prolonged glucose (*n* = 10), and prolonged PA with 5 mM glucose (*n* = 6). All data are presented as mean ± SEM, CI; Leak state complex I, CI + CII; Leak state complex I and complex II, ETS; Electron transfer system by uncoupling ATP synthase (complex V) with FCCP, LEAK; Leak state with oligomycin (state 4o), OXPHOS; Maximal respiration with 5 mM ADP (state 3), palmitic acid without glucose; Palmitic acid without glucose.

Tendency are presented as (*).

* Significantly different from regular glucose 5 mM, *p* < 0.05.

† Significantly different from acute regular palmitic acid (1.7 mM), *p* < 0.05.

‡ Significantly different from elevated palmitic acid (2.2 mM) in acute or prolonged intervention, *p* < 0.05.

** Significantly different from regular glucose 5 mM, *p* < 0.01.

*** Significantly different from regular glucose 5 mM, *p* < 0.001.

†† Significantly different from acute regular palmitic acid (1.7 mM) in presence of 5mM glucose, *p* < 0.01.

‡‡‡ Significantly different from elevated palmitic acid (2.2 mM) in acute or prolonged intervention, *p* < 0.001.

## 4 DISCUSSION

This is the first study to simultaneously assess mitochondrial respiration and H_2_O_2_ emission in skeletal muscle‐derived primary cultures of microvascular endothelial cells. The results show that both acute and prolonged (three‐day incubation) exposure of the endothelial cells to glucose and palmitic acid leads to mitochondrial dysfunction as assessed by altered mitochondrial respiration and H_2_O_2_/O_2_ emission. The following main observations were made: (i) in intact endothelial cells, presence of palmitic acid and glucose led to a markedly lower respiration and higher H_2_O_2_/O_2_ emission than presence of glucose; (ii) in intact and permeabilized cells, incubation for 3 days with elevated glucose levels led to a markedly lower mitochondrial respiration and a substantially higher H_2_O_2_/O_2_ emission than incubation for 3 days with regular glucose levels; (iii) in intact and permeabilized cells, incubation for 3 days with palmitic acid and glucose combined resulted in lower respiration and higher H_2_O_2_/O_2_ emission than incubation for 3 days with glucose alone.

### 4.1 Mitochondrial respiration and H_2_O_2_ emission with acute exposure to glucose and palmitic acid

Endothelial cells have been proposed to be sensitive to variations in nutrient availability (Caja & Enríquez, 2017). To assess how acute exposure of endothelial cells to glucose and palmitic acid affected respiration and H_2_O_2_ emission, measurements were conducted both in intact cells and permeabilized cells, tomore appropriately resemble in vivo conditions. The concentrations of substrates used were physiologically relevant, i.e. resembling concentrations seen in plasma in conditions of healthy versus lifestyle‐related disease (Abdelmagid et al., 2015; Bonora et al., 2001; Feng et al., 2017). Our results show that mitochondrial respiration and ROS formation in both intact and permeabilized cells were significantly lower and higher, respectively, in the presence of palmitic acid and glucose than with glucose alone and palmitic acid alone (Figure 4a and b). We are only aware of few studies which have examined the effect of acute substrate incubation, i.e. direct addition of substrate in the respirometer, on respiration and ROS in endothelial cells. In bovine aortic endothelial cells, passage 4–10 exposure to supraphysiological levels of glucose (30 mM) for 2 h was found to induce ROS formation, but without identification of mitochondria as a source of ROS (Nishikawa et al., 2000). By contrast, also in intact bovine aortic endothelial cells, passages 5–10, the presence of 5.5 mM glucose, 11 mM glucose, or 0.15 mM 18‐carbon fatty acids showed no effect on either respiration or H_2_O_2_ emission (Fink et al., 2012). The reason for the discrepancy in findings between studies is unclear, but one possible cause is the difference in cell origin, i.e. large vessel (aortic) endothelial cells versus primary microvascular endothelial cells derived from skeletal muscle. There is evidence in the literature that micro‐ and macrovascular cells differ in terms of gene expression (Minami & Aird, 2005) and metabolism (Lee et al., 2017). Although such a difference has not been directly demonstrated for skeletal muscle endothelial cells, it is likely that differences between micro‐and macrovascular cells are even greater in skeletal muscle, considering that changes in microvascular growth and energy demand can be markedly altered according to contractile activity (Egginton, 2009; Hellsten & Nyberg, 2016).

Moreover, the number of passages may well influence cell characteristics and it is well known that, in particular, endothelial cells are altered with the number of passages (Lee et al., 2009, 2010; Liao et al., 2014). The abovementioned study (Fink et al., 2012) on the effect of fatty acids also utilized a fatty acid concentration which was one tenth lower than used in the current study and also lower than regular plasma levels (Fink et al., 2012).

The observed ratio of leak to ETS respiration was higher with palmitic acid than with glucose (Table 1). While this alone does not necessarily indicate palmitate‐induced changes in mitochondrial coupling efficiency, it is worth noting that fatty acids have a well‐established role in inducing mitochondrial uncoupling in many other cell types including hepatocytes (Chan & Enns, 1979; Vaartjes & Bergh, 1978), myocytes (Boudina et al., 2005; Sparks et al., 2016) as well as white (Maassen et al., 2007) and brown (Fedorenko et al., 2012) adipocytes. The effect of palmitate on the relative capacity of OXPHOS and ETS is in accordance with the observation that their relative capacity varies between species and tissues (Pesta & Gnaiger, 2012), and to changes as a result of pathologies (Larsen et al., 2011; Pesta & Gnaiger, 2012).

### 4.2 Mitochondrial respiration and H_2_O_2_ emission with prolonged exposure to glucose and palmitic acid

To assess how prolonged exposure of the endothelial cells to glucose and palmitic acid affected mitochondrial respiration and H_2_O_2_ formation in the endothelial cells, measurements were conducted after the cells had been incubated for 3 days with regular or elevated glucose concentrations, and with regular or elevated palmitic acid concentrations combined with regular glucose. After incubation, measurements were conducted in the presence of 5 mM glucose.

The present study shows that prolonged exposure of endothelial cells to elevated glucose levels markedly decreases mitochondrial respiration and increases H_2_O_2_/O_2_ emission, both in intact and permeabilized cells. Similarly, prolonged incubation with a combination of regular palmitic acid and glucose levels led to lower respiration and higher ROS emission in both intact and permeabilized cells than with regular glucose alone. These findings on prolonged exposure to high substrate levels agrees well with the proposition that mitochondrial fuel overload with a concomitant low ATP demand maintains the proton gradient high, leading to inhibition of the electron transport chain and consequently enhanced ROS formation (Neufer, 2019). Notably, CS activity was not different between conditions (Figure 10) and that normalization of respiration to CS activity did not influence the results, suggesting that changes in mitochondrial content did not drive the observed changes (Figure 11b and c). The lack of change in CS activity also suggests that the relatively limited duration of the intervention did not induce a change at the protein level.

The flux control ratio in cells incubated with elevated glucose or combined glucose and regular palmitic acid showed, furthermore, that the LEAK state was similar to the ETS state (Table 1). This finding may indicate that endothelial cell mitochondria were fully uncoupled in the cells incubated with elevated substrate levels. The sole energy source during such conditions may thus be glycolysis.

Evidence in the literature for changes in mitochondrial respiration and mitochondria‐specific ROS formation in endothelial cells with prolonged exposure to metabolic substrates is limited, and results are conflicting. In HUVEC cells, 6 days of incubation with 150 µm palmitic acid was found to lower mitochondrial respiration and increase ROS formation (Broniarek et al., 2016). By contrast, a study on a hybrid endothelial cell line found no change in ROS formation and respiration with 48 h exposure of palmitic acid (Dymkowska et al., 2017). Nevertheless, the current findings suggest that in early passage muscle microvascular cells, prolonged exposure to high levels of metabolic substrates leads to reduced mitochondrial respiration and enhanced ROS emission. These data provide support for the notion that mitochondria may be central in microvascular endothelial dysfunction in lifestyle‐related disease (Fisher‐Wellman & Neufer, 2012; Neufer, 2019) and more specifically that impaired mitochondrial function is one of the mechanisms underlying the detrimental effects of hyperlipidemia and hyperglycemia in metabolic disease.

### 4.3 Characteristics of microvascular endothelial cell mitochondria

The present study shows that microvascular endothelial cells isolated from rodent skeletal muscle display measurable mitochondrial oxidative metabolism both in the presence of glucose and palmitic acid, in accordance with findings on large vessel endothelial cells, although endothelial cells are known to be primarily glycolytic (Caja & Enríquez, 2017; De Bock et al., 2013; Groschner et al., 2012; Li et al., 2019). Comparisons across different laboratories should be made with caution due to differences in laboratory protocols; however, the current magnitude of mitochondrial respiration is comparable to that reported for bovine aortic cells by use of a Seahorse respirometer (Fink et al., 2012). Mitochondrial ATP production has been shown to be of importance for endothelial function with regard to long‐term control of calcium release (Wilson et al., 2019) and inhibition of endothelial mitochondrial respiration abolishes cell proliferation (Coutelle et al., 2014; Olsen et al., 2020).

Moreover, the mitochondrial respiratory characteristics in the microvascular endothelial cells, as assessed by the SUIT protocol, appeared similar to that known from skeletal muscle and cardiac muscle mitochondria (Chrøis et al., 2020; Collins et al., 2019; Hey‐Mogensen et al., 2015; Larsen et al., 2011). The measured respiration per cell protein amount in the endothelial cells was estimated to be approximately 6 and 12% of that in human skeletal muscle and cardiac myocytes, respectively (Boyle et al., 2012; Larsen et al., 2012; Park et al., 2014) whereas the intrinsic respiratory capacity, i.e. respiration divided by citrate synthase activity, was 22.5–89 fold higher for rat endothelial cells compared with that of rat, mice, and human skeletal muscle tissue (Halling et al., 2019; Larsen et al., 2009, 2014, 2015). The markedly lower respiration per cell protein agrees well with previous reports of that endothelial cell mitochondria constitute 2–6% of the cell volume as opposed to 10–15% in skeletal muscle and 32% in cardiac myocytes (Kluge et al., 2013; Larsen et al., 2012).

### 4.4 Study limitations

The present study describes the influence of metabolic substrates on mitochondrial function in endothelial cells of microvascular origin. The study did not include a comparison with endothelial cells of macrovascular origin, thus a conclusion with regard to differences in response for cells from different vessel sizes cannot be made. Moreover, culture of cells will lead to a degree of alteration in phenotype and, although primary cell cultures were used at low passages, it is likely that culturing conditions would have led to some alteration in cell properties. Considering that in vivo cells are influenced by mechanical factors such as shear stress, the phenotype of the cultured cells in this study may therefore have differed somewhat from the origin. A limitation is also that the current study only included cells isolated from healthy animals and the influence of in vivo hyperglycemia and hyperlipidemia was not determined. Evaluation of the endothelial cells from animal models of metabolic disease as well as humans would clearly provide a valuable continuation to the current findings.

In conclusion, we present for the first time data from simultaneous measurements of mitochondrial respiration and H_2_O_2_ formation in skeletal muscle‐derived primary microvascular endothelial cells. Based on our findings, we conclude that prolonged incubation with excess substrate leads to a change in mitochondrial function in microvascular endothelial cells as evidenced by lower respiration and greater emission of H_2_O_2_. Thus in conditions of high plasma substrate availability, such as lifestyle‐related diseases, mitochondrial dysfunction in the microvascular endothelium may contribute importantly to endothelial dysfunction.

## CONFLICT OF INTEREST

No conflicts of interest, financial or otherwise, are declared by the authors.

## AUTHOR CONTRIBUTIONS

C.H. and Y.H. designed this experiment. K.O. optimized the cell‐method and assisted in experiment conduction. C.H. conducted all experiments, analysed data, and prepared figures. C.H., Y.H., and H.P. interpreted results of the experiments. C.H. and Y.H. drafted the manuscript. All authors revised the manuscript and approved the final version.

## Supporting information



Figure S1
