# Supplementary material for: High metabolic substrate load induces mitochondrial dysfunction in rat skeletal muscle microvascular endothelial cells
Source: Physiol Rep. 2021 Jul 20;9(14):e14855. doi: 10.14814/phy2.14855 (PMC8290479; doi:10.14814/phy2.14855)
Supplement: Supplementary file 1 — Figure S1 [file PHY2-9-e14855-s001.docx]

# **Supplementary**

#
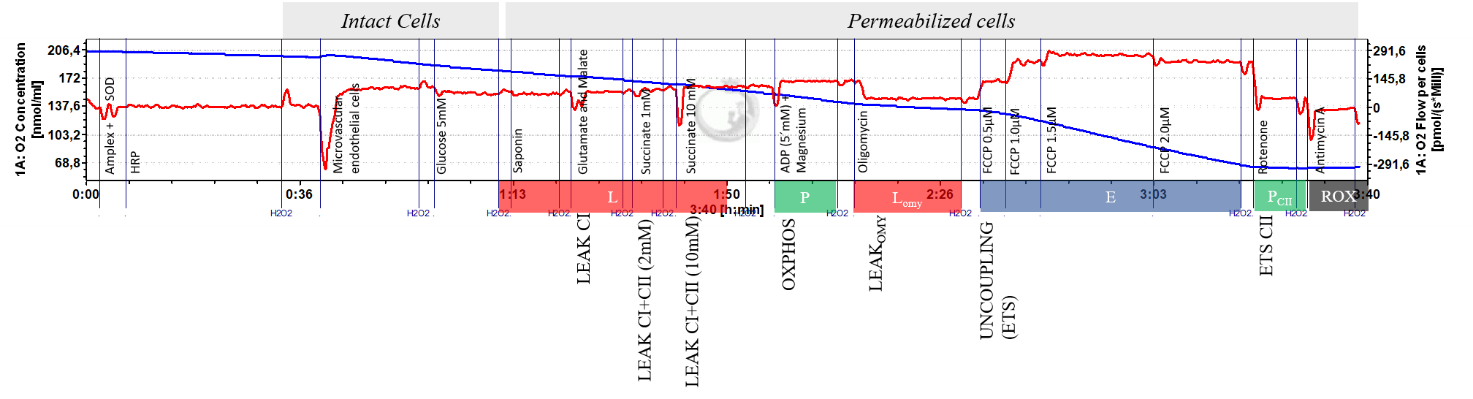


Fig.1S. A representative trace of one mitochondrial analysis and the Substrate-Uncoupler-Inhibitor Titration (SUIT) protocol.
ADP; Adenosine disphosphat, Amplex; Amplex UltraRed, E; is the respiratory electron transfer pathway capacity of the mitochondria in an uncoupled state, FCCP; Carbonyl cyanide-p-trifluoromethoxyphenylhydrazone, HRP; Horse Radish Peroxidase, H2O2; hydrogen peroxides, L; Leak state without exogenous ADP or with inhibition with oligoycin and ATP-synthase inhibitor, P; is the respiratory capacity of mitochondria in the ADP-activated state of [oxidative phosphorylation](https://wiki.oroboros.at/index.php/Oxidative_phosphorylation), ROX; is residual oxygen consumption, respiration in this state is due to oxidative side reactions remaining after inhibition of the electron transfer, SOD; superoxide dismutase.
